# Localization of Secondary Metabolites in Relict Gymnosperms of the Genus *Sequoia* In Vivo and in Cell Cultures In Vitro, and the Biological Activity of Their Extracts

**DOI:** 10.3390/life14121694

**Published:** 2024-12-20

**Authors:** Rima N. Kirakosyan, Elena A. Kalasnikova, Elizaveta A. Bolotina, Abdulrahman Saleh, Anastasiya A. Balakina, Svetlana M. Zaytseva

**Affiliations:** 1 Department of Biotechnology, Russian State Agrarian University—Moscow Timiryazev Agricultural Academy, Timiryazevskaya Street 49, Moscow 127434, Russia; kalash0407@mail.ru (E.A.K.); mouse-liza@mail.ru (E.A.B.); abdulrahman1996nez@gmail.com (A.S.); 2 Federal Research Center of Problems of Chemical Physics and Medicinal Chemistry, Russian Academy of Science, Ac. Semenov Avenue 1, Moscow Region, Chernogolovka, Moscow 142432, Russia; balakina@icp.ac.ru

**Keywords:** coast redwood (*Sequoia sempervirens*), in vitro plant cell cultures, phytotoxicity of extracts, cytotoxicity of extracts, photoperiodism, localization, secondary metabolites, phenolic compounds, terpenoids

## Abstract

In order to scientifically search for new sources of secondary metabolites with valuable qualities for phytopharmacognosy, tasks requiring a step-by-step solution were set. The primary task is the development of technologies for obtaining in vitro highly productive biomass of cells of relict gymnosperms of the genus *Sequoia*, capable of accumulating various classes of secondary metabolites. The study of the accumulation and localization of secondary metabolites allowed us to evaluate the biological activity and cytotoxicity of in vitro *Sequoia* cultures. In our study, histochemical methods were used to determine the localization of secondary compounds (phenolic and terpenoid in nature) in plant tissues. Secondary metabolites—polyphenols, catechins, and terpenoids—are mainly localized in the epidermal, parenchymal, and conductive tissues of *Sequoia* leaves and stems. In callus and suspension cultures of *Sequoia*, secondary metabolites were localized in cell walls and vacuoles. The mineral composition of the nutrient medium (MS and WPM), the light source (photoperiod), and the endogenous content of polyphenols in the primary explant influenced the initiation and growth characteristics of the in vitro culture of *Sequoia* plants. Inhibition of growth in suspension cultures on the WPM nutrient medium was noted. The cultivation of *Sequoia* cell lines at a 16 h photoperiod stimulated the formation of polyphenols but had a negative effect on the growth of callus cultures. Extractive substances obtained from intact and callus tissues of evergreen *Sequoia* demonstrate high biological (fungicidal) activity and cytotoxicity. The inhibitory effect on *Fusarium oxisporum* was noted when 200 mg/L of *Sequoia* extract was added to the nutrient medium. Extracts of redwood callus cultures were low in toxicity to normal FetMSC cells but inhibited the growth of lines of “immortal” cervical HeLa cancer cells and human glioblastoma A172. Intact tissues of *Sequoia* plants and cell cultures initiated from them in vitro are producers of secondary metabolites with high biological activity.

## 1. Introduction

Plants that produce useful secondary metabolites typically have a narrow range of growth in highly specialized climatic conditions, in contrast to the main food crops [1]. Variability in the accumulation of compounds useful for the pharmaceutical industry is a result of the impact of exogenous environmental conditions (such as pathogen attack, allelopathic interaction, soil mineral composition, illumination, etc.) on plants’ secondary metabolism [2,3]. Natural populations have been reduced, and some species of medicinal plants have gone extinct as a result of unchecked raw material gathering and human pressure. To maintain the biodiversity of rare, therapeutic plant species, it is recommended to employ biotechnology techniques and establish in vitro genetic banks [4,5]. As is known, compounds of phenolic and terpenoid natures are the most common representatives of secondary plant metabolism [6,7]. They are biologically active substances with a rich spectrum of pharmacological action, including antibacterial, antiviral, and antitumor properties [8]. In addition, secondary compounds are metabolites that are physiologically active mediators and signaling molecules in plant life. They participate in respiration, photosynthesis, reproduction, plant growth, and development, as well as in the process of dedifferentiation in vitro [9,10,11,12].

Currently, many thousands of plants are known, in various organs of which valuable metabolites of a secondary nature with high biological activity accumulate [13,14]. For example, among gymnosperms, representatives of the genus *Taxus* occupy a special place in the biosynthesis of secondary metabolites due to their unique ability to synthesize species-specific cytotoxic substances. As a rule, such plants are relict centenarians, characterized by an extremely slow growth rate, unsatisfactory reproduction, and low viability in in vitro culture [15,16]. Therefore, the search for alternative, safe, closely related plant sources rich in broad-spectrum polyphenols remains an urgent area of research. One of the promising closely related objects, whose biomass contains polyphenols, is the evergreen *Sequoia* (*Sequoia sempervirens* (D.Don) Endl.), a relict coniferous tree growing in a narrow range [17,18]. *Sequoia* species are the tallest members of the plant Kingdom; individual specimens can reach heights of over 100 m, and some of the live trees are thought to be over 2500 years old [19,20]. Researchers believe that some gymnosperms’ remarkable longevity and tolerance to stressors, including pathogenic ones, are due to the active secondary biosynthesis and accumulation of polyphenols in their cells [21,22].

Since *Sequoia* belongs to relict species and has a limited, highly specialized distribution area in nature, technologies that allow the renewal of *Sequoia* plant material without its removal from the natural environment are of great practical importance. In addition to clonal micropropagation, such technologies include in vitro tissue culture methods that allow obtaining raw materials with high biological activity in a short time. An analysis of the literature data shows that most studies are aimed at developing methods that enhance natural regeneration based on the morpho-physiological characteristics of *Sequoia* [17,23]. As for studies on the production of callus and suspension cultures of plants of the genus *Sequoia* as potential sources of valuable secondary metabolites, there are few [24,25]. Studies on the formation and localization of secondary compounds in the tissues of intact plants in vivo and dedifferentiated cells in vitro have not previously been conducted. Based on the above, the purpose of this work was to develop a technology for obtaining in vitro highly productive biomass of cells of relict gymnosperms of the genus *Sequoia* and to study the localization of secondary metabolites and their biological activity.

## 2. Materials and Methods

### 2.1. The Object of Research

**Figure 1:**
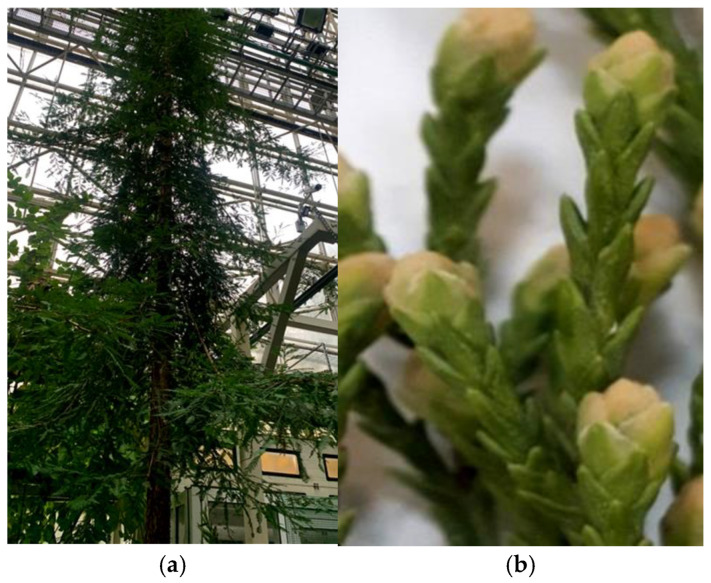
Appearance of evergreen *Sequoia* plants (**a**) in a greenhouse and (**b**) cuttings used as explants for in vitro cultivation.

The object of the study was cuttings of the coast redwood (*Sequoia sempervirens* (D.Don) Endl.) collected from adult trees growing in the Moscow region in a greenhouse at the N.V. Tsitsin Main Botanical Garden of the Russian Academy of Sciences, during the period from November to December, from the first-year growth shoots (Figure 1). Before being introduced into in vitro culture, the primary explants underwent a stepwise sterilization process. Initially, the cuttings were washed in a soapy solution for 10 min, then rinsed with running water for 2 min and treated with a weak solution of potassium permanganate for 10 min. Following this, in a laminar flow hood, the cuttings were sterilized with a 0.1% solution of mercuric chloride (HgCl_2_) for 18 min and washed three times with sterile distilled water. The cuttings were then sliced with a scalpel into segments measuring 1.5–2 cm in length, retaining 7–10 needles, and placed in biological test tubes containing an agarized nutrient medium with mineral salts according to the Murashige and Skoog (MS) formulation [26] or Woody Plant Medium (WPM) [27], auxins, and cytokinins, represented by indole-3-butyric acid (IBA) (Merck, Darmstadt, Germany) at a concentration of 2.5 mg/L or 2,4-dichlorophenoxyacetic acid (2,4-D) (Merck, Darmstadt, Germany) at a concentration of 2.5 mg/L combined with 6-benzylaminopurine (BAP) (Merck, Darmstadt, Germany) at 2.5 mg/L, 2% sucrose, and 0.7% agar. The pH of the medium was adjusted to 5.6–5.8 before autoclaving. The test tubes were placed in a light room on shelves under white linear fluorescent lamps, with an intensity of 150 µmol/m^2^·s, and cultivated at a temperature of 23 ± 1 °C with a 16 h photoperiod.

The culture was subcultured every 6 weeks, taking into account the biometric and morphological parameters of the formed microshoots and callus aggregates. All work on the sterilization of primary explants, their introduction into in vitro culture, and the study of morphogenetic potential was conducted under aseptic conditions in a laminar flow hood (BIOBASE BBS-H1800(X), Shandong, China).

### 2.2. Obtaining and Cultivating Callus Tissue

Callus tissue was obtained from the needles as well as from the internodes of shoots isolated from intact *Sequoia* plants collected from adult trees growing in the Moscow region in a greenhouse at the N.V. Tsitsin Main Botanical Garden of the Russian Academy of Sciences, during the period from November to December, from first-year growth shoots. A multi-step sterilization was performed beforehand. Initially, the cuttings were washed in a soapy solution for 10 min, rinsed with running water for 2 min, and treated with a weak solution of potassium permanganate for 10 min. Following this, in a laminar flow hood, the cuttings were sterilized with a 0.1% solution of mercuric chloride (HgCl_2_) for 18 min and washed three times with sterile distilled water. The cuttings were then sliced with a scalpel into segments measuring 0.8–1.0 cm in length, retaining 3–5 needles, and placed in 90 mm Petri dishes. The explants were cultivated on a nutrient medium containing mineral salts according to the Murashige and Skoog (MS) formulation or Woody Plant Medium (WPM), supplemented with growth regulators at various concentrations—2,4-dichlorophenoxyacetic acid (2,4-D) (Merck, Darmstadt, Germany) at a concentration of 2.5 mg/L combined with 6-benzylaminopurine (BAP) (Merck, Darmstadt, Germany) at 2.5 mg/L, 2% sucrose, and 0.7% agar. The pH in all variants was set to 5.8.

Callus culture was carried out in 90 mm Petri dishes. The Petri dishes with plant material were divided into two groups: one group was placed on shelves under white linear fluorescent lamps (OSRAM AG, Munich, Germany) (light intensity 150 µmol/m^2^·s) and cultivated at a temperature of 23 ± 1 °C with a 16 h photoperiod, while the second group was grown without light access. The transfer of callus tissue to a fresh nutrient medium was carried out once every 4 weeks. During this process, the time and intensity of callus formation, as well as its consistency and color, were monitored.

To characterize the callus tissue, indicators such as the growth index (*I*) and specific growth rate (µ) were also taken into account and calculated using Formulas (1) and (2).

(1) $$I=\frac{X_{\max}-X_{0}}{X_{0}},$$

where *X*_max_ and *X*_0_ are the maximum and initial values of the callus tissue diameter, respectively (in cm).

To determine the specific growth rate (µ), the following formula was used:

(2) $$µ=\frac{\ln X_{2}-\ln X_{1}}{t_{2}-t_{1}},$$

where *X*_2_ and *X*_1_ are the content of dry biomass in 1 L of medium (in mg) at times *t*_2_ and *t*_1_ (in days), respectively.

### 2.3. Obtaining and Cultivating Suspension Cell Cultures

The suspension cell culture was obtained from homogeneous callus tissue of *Sequoia* after the third passage, initiated from both intact shoot segments and from the needles of sterile microshoots in vitro, characterized by a high growth index.

The obtaining and cultivation of the suspension culture were conducted in a liquid nutrient medium containing mineral salts according to the Murashige and Skoog (MS) formulation or Woody Plant Medium (WPM), as well as auxins 2,4-D at a concentration of 2.5 mg/L combined with BAP at 2.5 mg/L and 2% sucrose. The pH in all variants was set to 5.8. The suspension culture was cultivated in 100 and 250 mL round and conical flasks on a shaker (CRYSTY) in the dark at a temperature of 23 ± 1 °C and a rotation speed of 100 rpm. The flasks, nutrient media, and accompanying materials used in the work were sterilized in an autoclave (GK-100-3, Moscow, Russia) for 20 min at a pressure of 1 atm. Cell volumes of 1 mL and 2 mL were used as inoculum.

### 2.4. Subculturing Cycle for Suspension Culture

The subculturing cycle for the suspension culture in flasks lasted 14 days. The following indicators were used to characterize the suspension cell culture: growth index (*I*) and specific growth rate (µ), which were calculated using Formulas (1) and (2).

### 2.5. Localization of Secondary Compounds

The localization of secondary compounds in callus tissue was studied using histochemical methods. Localization was determined on living temporary preparations: the sum of phenolic compounds was stained with 0.08% Fast Blue reagent [28,29,30,31,32], and flavans (catechins and proanthocyanidins) were reacted with vanillin reagent in hydrochloric acid vapors. The localization of terpenoids was determined using NADH reagent [33]. Preparations were examined under a Zeiss light microscope (Oberkochen, Germany).

### 2.6. Investigation of the Fungicidal Activity of Extracts

For primary screening of fungicidal activity, concentrated ethanol extracts obtained from raw biomass of shoots and callus tissue of *S. sempervirens* weighing 1 g were used. All experiments were conducted on pure cultures of fungi of the genus *Fusarium oxysporum* L. This strain was isolated and identified by staff members of the Mycology Laboratory of the Institute of Phytopathology of the Russian Academy of Sciences. Live cultures of *Fusarium oxysporum* L. fungi, which had been stored for a long time in a refrigerator at +4 °C, were initially propagated on a nutrient medium containing mineral salts according to the MS formulation, without plant hormones. Fungi were grown in Petri dishes in a light room at a temperature of 25 °C, with a 16 h photoperiod and light intensity of 3000 lux. No subculturing was performed.

The dry plant extract residue was obtained from concentrated ethanol extracts of intact shoots of *Sequoia* and initiated from them third-passage callus tissue. For this, a sample of 1 g of plant material was extracted with 10 mL of 96% ethanol for 1 h at a temperature of 37 ± 1 °C, filtered through a paper filter (blue stripe), evaporated to dryness, and weighed to determine the exact mass. A sample of the lyophilized dry extract residue was dissolved in DMSO and added to the nutrient medium after autoclaving. The extract concentrations were 50, 100, 150, 200, 250, 300, 350, 400, 450, and 500 mg/L. The control was the medium without extract, as well as the pure solvent (DMSO). The fungicidal activity of plant extracts was determined by the growth of fungal mycelium. To do this, the diameter of the fungus was measured in two planes during the 21-day cultivation period [34].

### 2.7. Cultivation of Mammalian Cells

The following cell cultures obtained from the collection of the Institute of Cytology of the Russian Academy of Sciences (St. Petersburg, Russia) were used in the study: FetMSC (human embryonic mesenchymal stem cells), A-172 (human glioblastoma), HeLa (human cervical adenocarcinoma, clone M), and HepG2 (human hepatocellular carcinoma). Cells were cultured using standard procedures in an atmosphere of 5% CO_2_ at a temperature of 37 °C ± 1 °C. A-172 cells were cultured in DMEM medium (“PanEco”, Moscow, Russia) supplemented with 10% fetal calf serum (“BioWest”, Nuaillé, France), penicillin (50 U/mL), and streptomycin (50 mg/mL). HepG2 and HeLa cells were cultured in EMEM medium (“PanEco”, Moscow, Russia) supplemented with 10% fetal calf serum, penicillin (50 U/mL), and streptomycin (50 mg/mL). FetMSC cells were cultured in F12/DMEM medium (“PanEco”, Moscow, Russia) supplemented with 10% fetal calf serum (“BioWest”, Nuaillé, France), 50 U/mL penicillin, and 50 mg/mL streptomycin.

### 2.8. Investigation of Extract Cytotoxicity

Extracts were obtained from 1 g of raw biomass of third-passage callus tissue of *S. sempervirens*. For this, a sample of 1 g of plant material was extracted with 10 mL of 96% ethanol for 1 h at a temperature of 37 ± 1 °C, filtered through a paper filter (blue stripe), evaporated to dryness, and weighed to determine the exact mass. The cytotoxic properties of the extracts were studied using the MTT assay. Cells were seeded in 96-well culture plates at a concentration of 5 × 10^4^ cells/mL for HeLa and A-172, 7 × 10^4^ cells/mL for HepG2, and 1 × 10^5^ cells/mL for FetMSC cells. Lyophilized samples were dissolved in 96% ethanol to a final concentration of 100 mg/mL based on dry weight. To 990 µL of the culture medium, 10 µL of the alcoholic solution was added to achieve a final concentration of 1000 µg/mL. Extracts were added to the nutrient medium 24 h after cultivation. Cytotoxicity of the extracts was evaluated after 72 h of exposure at concentrations ranging from 7.5 to 1000 µg/mL. At the end of the experiment, cells were incubated for 3 h in the presence of 3-(4,5-dimethylthiazol-2-yl)-2,5-diphenyl-2H-tetrazolium bromide (MTT, DiaM, Moscow, Russia) at a concentration of 0.5 mg/mL. The resulting formazan crystals were dissolved in 100% DMSO.

### 2.9. Optical Density Measurement

Optical density was measured at a wavelength of 570 nm and a background wavelength of 620 nm using a multifunctional plate reader Spark 10M (Tecan, Männedorf, Switzerland). The cytotoxicity index (IC50) was determined from dose-response curves using median effect analysis.

### 2.10. Statistical Data Processing

The mean values of all data were calculated using Microsoft Office Excel 2013 packages. Analysis of variance (ANOVA) was performed using Statistica, version 10.0, and means were compared using Duncan’s multiple range test at a significance level of α = 0.05.

## 3. Results

### 3.1. Localization of Secondary Metabolites in Intact Sequoia Plants

Secondary metabolites, particularly phenolic compounds (PCs), can significantly influence callus formation and the growth of cultures in vitro [35]. Therefore, it is essential to establish a correlation between the formation of callus tissue and the distribution of secondary metabolites, including polyphenols. To achieve this, we aimed to identify the areas of maximum localization of these secondary compounds throughout the explant.

**Figure 2:**
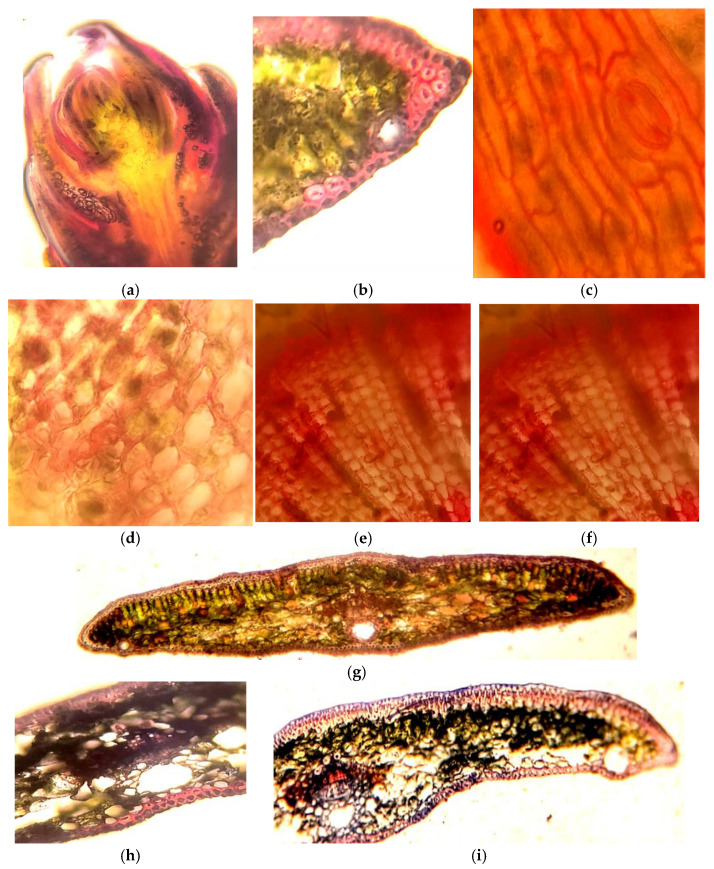
Localization of phenolic compounds in (**a**) meristematic, protective (**b**,**c**), and conducting (**d**–**f**) tissues of *Sequoia* used as explants for initiating callus cultures. (Reaction with vanillin reagent for flavonoids (**a**,**c**) and total phenolic content with Fast Blue reagent (**b**)). Reaction with NADH reagent for the presence of terpenoids (**g**–**i**).

Our histochemical studies demonstrate a specific localization of secondary metabolites of phenolic and terpenoid nature in the leaf blade (needles) and stem. Generally, these compounds are predominantly located in the protective and conductive tissues, with their presence also noted in parenchymal cells. The results show a significant presence of secondary metabolites in the conducting tissues of the *Sequoia* stem, particularly within the cell walls of the xylem vessels and the phloem. The most pronounced reaction was observed for flavonoid compounds, especially in the cells of the heartwood (Figure 2a,b).

In the needles, the parenchymatous sheath surrounding the vascular bundles and the albuminous cells were particularly rich in these compounds. Additionally, the localization of polyphenols was observed in the cell walls, intercellular spaces, and specialized phenol-storing epiblastic cells, appearing as amorphous substances or granulated inclusions of varying aggregation in mesophyll cells and guard cells (Figure 2c).

The reaction with vanillin reagent on flavonoids in the studied explants is analogous to the response of cells to the Fast Blue reagent, which indicates the total content of soluble phenolic compounds. This suggests that the phenolic complex in the explant of *Sequoia* sempervirens is dominated by flavonoid compounds, particularly evident in the meristematic tissues.

It should be noted that in the studied tissues of the explants, the reaction to terpenoids is also most pronounced in the conducting structures, the parenchymatous sheath of the vascular bundles, and the albuminous cells (Figure 2h,i). This observation correlates with the staining using the Fast Blue reagent, which indicates the total content of soluble phenolic compounds.

### 3.2. Obtaining and Cultivating Callus Tissue

**Figure 3:**
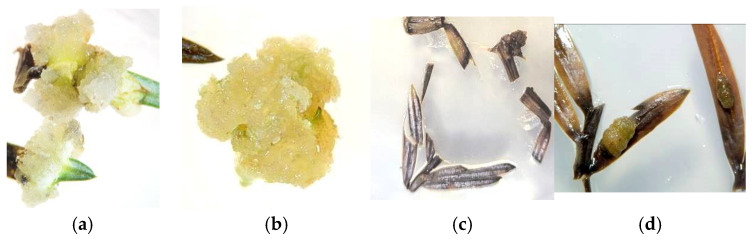
Formation of primary callus tissue at wound sites: cultivation of explants in darkness on WPM medium (**a**) and MS medium (**b**); cultivation of explants in the presence of light under a 16 h photoperiod on WPM medium (**c**) and MS medium (**d**).

**Figure 4:**
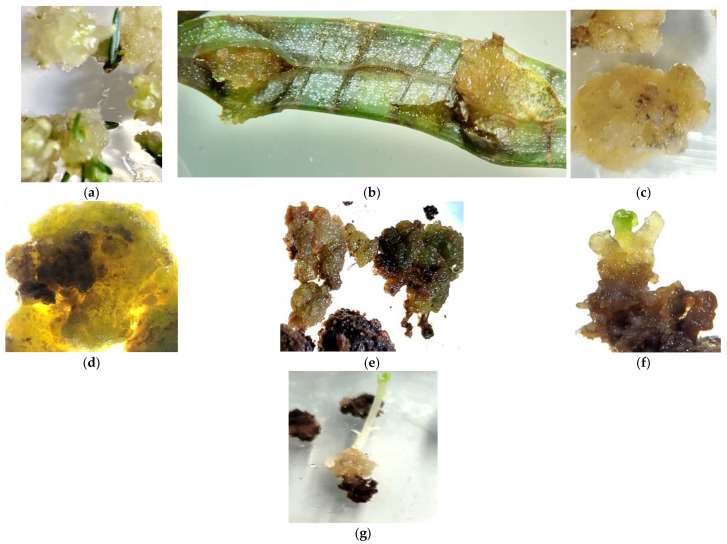
Formation of callus tissue on nutrient media with varying mineral content and under different light conditions: without light access on MS medium (**a**) and WPM medium in darkness (**b**); callus tissue from the third passage cultivated in darkness (**c**); callus tissue from the third and fourth passages (**d**,**e**) cultivated under a 16 h photoperiod inducing singular morphogenesis (**f**,**g**).

**Figure 5:**
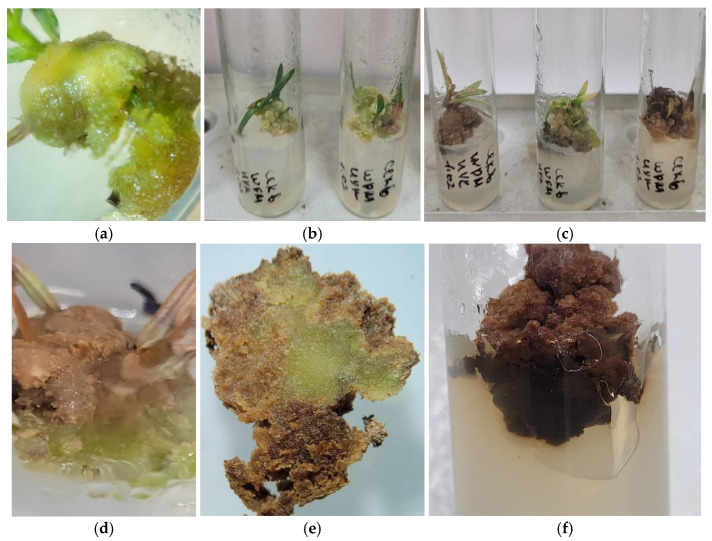
Initiation of callus tissue from *Sequoia sempervirens* (D.Don) Endl. on MS media (**a**,**d**,**e**) and WPM (**b**,**c**,**f**), and its subsequent cultivation for four (**c**,**d**) and twelve (**f**) months under light in vertical tubes.

The conducted studies have revealed several patterns in the formation of callus tissue:

1. In all variations, proliferation of callus cells was observed at the sites of cuts and injuries.
2. The onset of callus formation was noted between the 14th and 17th days of cultivation in darkness.
3. Generally, the callus tissue was of medium density and had a white-yellow color.
4. The formation of primary callus tissue occurred over the entire surface of the shoot segment and also from the mesophyll of the leaf blade located near the central vein (Figure 3a,b and Figure 4a–c).
5. When cultivating primary explants (shoot segments) horizontally in light, callus formation was inhibited, and only in rare cases on days 30 to 40 was dark brown callus with green inclusions observed (Figure 3c,d).
6. When cultivating primary explants (shoot segments) vertically in light, callus formation of yellow-green color was observed in the zone of contact between the shoot and the nutrient medium by days 17 to 20 (Figure 5a,b).

Experiments have experimentally established that the conditions of cultivation, such as the type of vessel (Petri dishes or tubes) and lighting conditions (in light or darkness), significantly influence the intensity of callus tissue formation, its consistency, and its color (Figure 4 and Figure 5). For instance, the horizontal cultivation of *Sequoia* sempervirens explants in Petri dishes on MS and WPM nutrient media in darkness led to the formation of callus tissue that was white and light-yellow in color with medium density. Notably, the onset of callus formation was already observed on the 14th day of cultivation.

For explants placed on WPM, primary callus formation was primarily characterized on the leaf blade, predominantly in the mesophyll area near the midrib, while for explants placed on MS medium, it was noted on the stem in the internodes and meristems (Figure 4a,b). The callus initiated in darkness during cultivation exhibited intense growth and a light coloration (Figure 4c).

### 3.3. Influence of Light Conditions on Callus Formation in Sequoia sempervirens

A different scenario was observed when cultivating *Sequoia* sempervirens explants in Petri dishes on MS and WPM media under light conditions. In this cultivation variant, the callus tissue exhibited a loose consistency and formed a yellow-brown color (Figure 4d,e. However, it is important to note that the percentage of callus formation did not exceed 10%, and the onset of callusogenesis was only observed between the 30th and 40th days. The initiated callus showed weak growth intensity and viability.

During the cultivation process, the tissue turned dark brown with small green elements, and growth processes were completely halted, leading to partial tissue death. Despite the low viability, after three or four cycles of cultivation, a few poorly defined meristematic foci were noted (Figure 4e,f), from which it was not possible to induce fully functional regenerant plants. Therefore, obtaining and cultivating callus tissue under light conditions proved to be impractical, and in subsequent experiments, the callus tissue was grown exclusively in darkness.

### 3.4. Effects of Vertical Cultivation on Callus Formation in Sequoia sempervirens

However, it should be noted that when cultivating *Sequoia* sempervirens explants vertically in tubes on WPM and MS media in the presence of light, significant changes in callus tissue formation were observed. In this variant, by the 17th to 20th day of cultivation, 100% callus formation was noted, which exhibited a yellowish and beige-green color, high growth rates, and remarkable viability (Figure 5). During the subsequent four cycles of cultivation, the upper part of the callus tissue took on a brownish hue, while the lower part remained light-yellow with green inclusions (Figure 5e,f). However, when the callus tissue was cultivated for 12 cycles, a gradual browning was observed, yet the callus retained a high growth rate, with light areas forming in the central part (Figure 5d,e).

### 3.5. Establishment and Cultivation of Suspension Culture of Sequoia sempervirens

**Figure 6:**
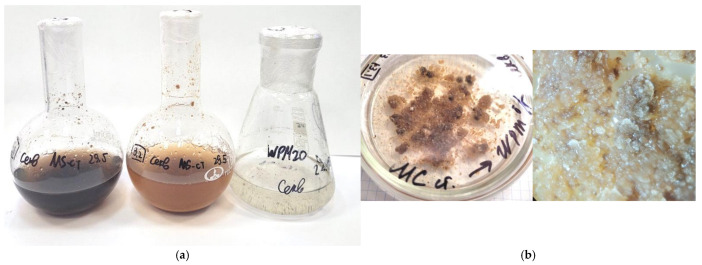
Suspension cultures of *Sequoia sempervirens* (D.Don) Endl. cultivated on liquid nutrient media MS (**a**) and WPM (**a**) for 6 months, as well as their subsequent transfer to solid nutrient media according to the WPM formulation (**b**).

The mineral composition of the culture medium plays a crucial role in the establishment and cultivation of the suspension culture of S. sempervirens. When using media containing mineral salts according to the MS and WPM recipes with a hormonal composition similar to solid agar media, the influence of the mineral composition on the growth characteristics of the suspension was observed (Figure 6a). During the first and second cycles of culturing the cell suspension of *Sequoia* in media containing mineral salts according to the MS and WPM recipes in darkness, medium- or large-aggregated cultures formed. The best growth index (*I* = 12) was achieved using MS medium, where the suspension culture exhibited a light-yellow color (Figure 6). In contrast, when using WPM medium, the suspension culture exhibited slower growth (*I* = 5.8) and formed large dense aggregates of dark brown color. The subsequent passage of such a suspension culture onto fresh WPM medium did not normalize the growth processes (Table 1).

However, transferring it to a MS medium allowed the growth of the suspension culture to resume.

### 3.6. Localization of Secondary Metabolites in Sequoia In Vitro Cultures

Further studies focused on exploring the localization characteristics of active secondary metabolites—specifically, polyphenolic and terpenoid compounds—which are integral to understanding the physiological and biochemical status of callus and suspension cultures influenced by various factors.

**Figure 7:**
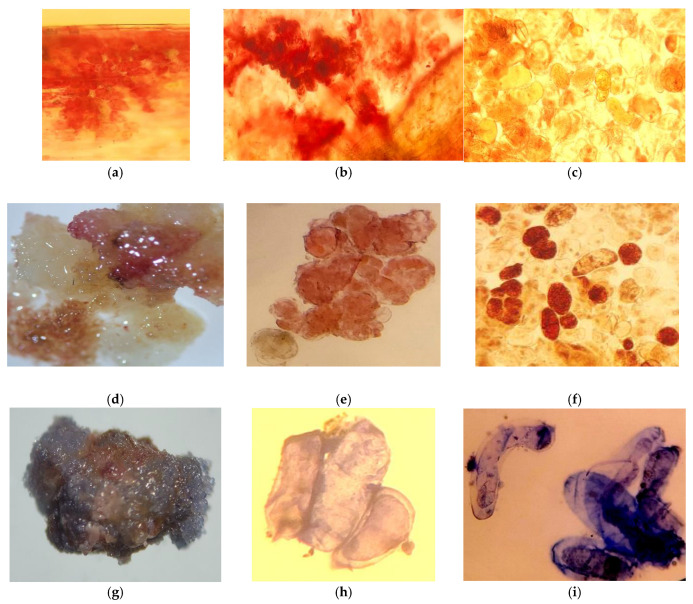
Localization of phenolic compounds during the initiation of callus tissue on the explant (**a**,**b**), in the cells of primary callus tissue (**c**), and in the third passage callus of *Sequoia* cultivated in the absence of light (**d**–**f**). Reaction to the sum of soluble phenolic compounds with Fast Blue reagent (**a**,**b**,**c**,**f**) and with vanillin reagent for the localization of flavonoids (**d**,**e**). Reaction with NADI reagent for the presence of terpenoids in the primary callus (**g**,**h**) and third passage (**i**).

**Figure 8:**
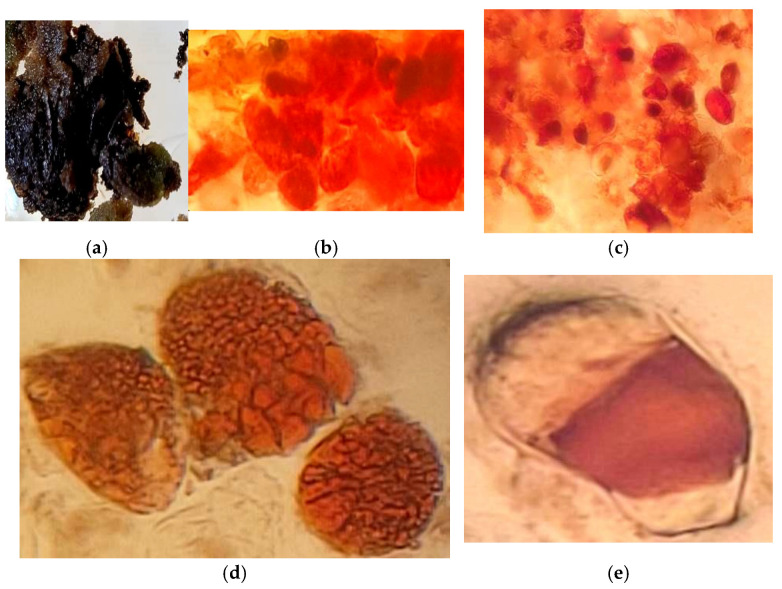
Callus tissue obtained in the presence of light (**a**), localization of phenolic compounds during callus initiation on the explant (**b**), and in the poorly viable callus tissue after 3 months of subculturing in the presence of light (**c**–**e**). Reaction to the sum of soluble phenolic compounds with Fast Blue reagent (**d**) and with vanillin reagent for the localization of flavonoids (**b**,**c**,**e**).

Our research demonstrated that even in the early stages of cultivation, under different lighting conditions, the callus tissues of *Sequoia* varied not only in growth but also in biosynthetic activity, as confirmed by histochemical analyses. At the boundary of callus tissue initiation on the primary explant, a protective layer of cells containing phenolic compounds was formed (Figure 7a,b). During the first passages, there was a marked formation of specialized storage cells (epiblasts), which accumulated phenolic compounds as fine- and coarse-granulated inclusions. Large accumulations of epiblasts were observed in both the central and peripheral regions of the callus tissue (Figure 7 and Figure 8). Such accumulations were characteristic of callus tissue initiated in both light and dark conditions. However, for cultures grown in light, the reaction to polyphenols was widespread and exhibited greater intensity, particularly regarding flavonoid compounds (Figure 8b–d). Terpenoid compounds were also detected in significant quantities in the cells of the primary callus, and their staining intensity remained unchanged with successive passages (Figure 7g–i).

**Figure 9:**
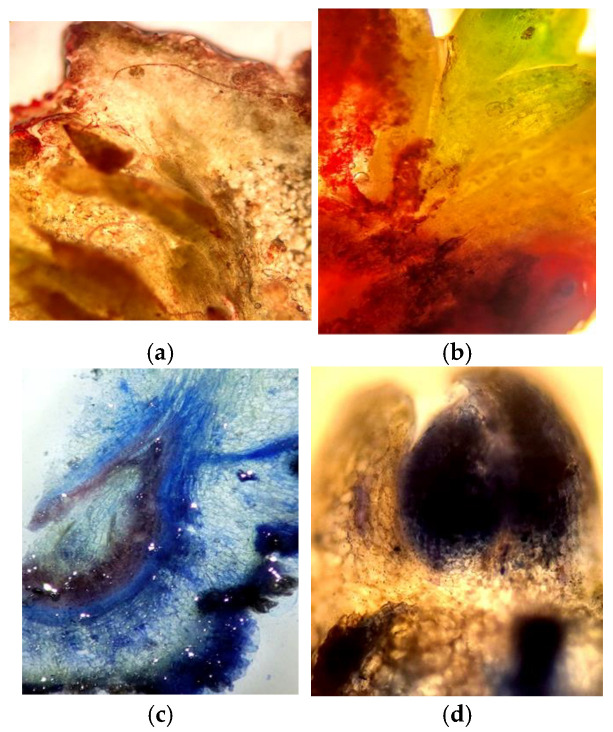
Localization of secondary metabolites in the morphogenic callus of *Sequoia*. Reaction for the total soluble phenolic compounds with Fast Blue reagent (**a**) and vanillin reagent for the localization of flavonoids (**b**). Reaction with NADI reagent for the presence of terpenoids (**c**,**d**).

In callus tissue initiated and cultivated for more than three passages under light conditions, meristematic zones with increased metabolic activity concerning the studied secondary metabolites were observed. The morphogenic callus was characterized by a high density of phenol-storing cells (epiblasts) in the basal part of the callus conglomerate, which contained flavonoid compounds of varying degrees of aggregation (Figure 9). The meristematic zones were also rich in phenolic and terpenoid compounds, which densely surrounded the differentiation zone (Figure 9c).

### 3.7. Suspension Culture of Sequoia and Studies on Fungicidal and Cytotoxic Activity of Extracts

#### 3.7.1. Formation of Secondary Metabolites in Suspension Culture

**Figure 10:**
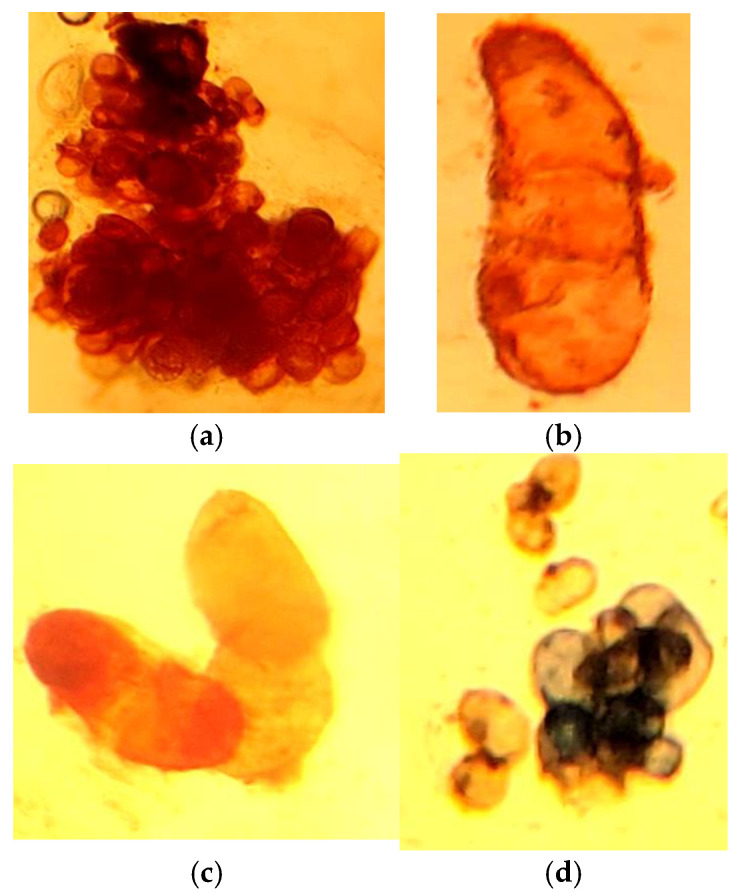
Localization of phenolic compounds (**a**–**c**) and terpenoids (**d**) in suspension culture of *Sequoia*. Reaction for the total soluble phenolic compounds with Fast Blue reagent (**a**,**b**) and vanillin reagent for the localization of flavonoids (**c**). Reaction with NADI reagent for the presence of terpenoids (**d**).

In the suspension culture of *Sequoia*, the formation of individual or cell aggregates was noted, in which secondary metabolites accumulated. Polyphenols and terpenoids were localized in the cell walls and vacuoles as amorphous substances and granulated inclusions of varying degrees of aggregation (Figure 10).

#### 3.7.2. Investigation of Fungicidal Activity of Extracts

To analyze the fungicidal activity of complex extracts from the biomass obtained from the shoots of *Sequoia* during the winter growing season and from callus tissue in the third cultivation cycle, we utilized the mycelium of the fungus *Fusarium oxisporum* L. This fungus was cultivated on nutrient media containing various concentrations of the studied extracts and grown for 21 days. The diameter of the fungus was measured in two planes over time.

**Figure 11:**
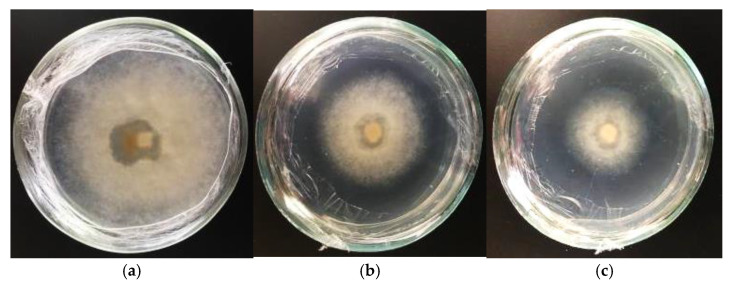
Fungicidal activity. Changes in the growth of fungal mycelium under the influence of extracts obtained from intact plants (**a**,**b**) and sequoia callus tissue (**c**) at a concentration of 200 mg/L extract, 400 mg/L extract and 500 mg/L extract, respectively.

The results of the fungicidal activity studies of the extract samples against *F. oxisporum* at the tested concentrations are presented in Table 2. An inhibitory effect against *F. oxisporum* was observed with the addition of 200 mg/L of the extract to the nutrient medium, with the effect strengthening as the concentration increased to 350 mg/L and higher. In these cases, the inhibition of fungal mycelium growth exceeded 60% compared to the control. However, the fungicidal activity of extracts obtained from the shoots of intact plants and from callus tissue differed. Notably, extracts from shoots had an inhibitory effect 1.5 times greater than that of extracts from callus tissue. The use of higher concentrations of the studied extracts enhanced the inhibitory activity, but complete suppression of *F. oxisporum* mycelium growth was not observed in any case (Figure 11).

#### 3.7.3. Investigation of Cytotoxic Effects of Extracts

**Figure 12:**
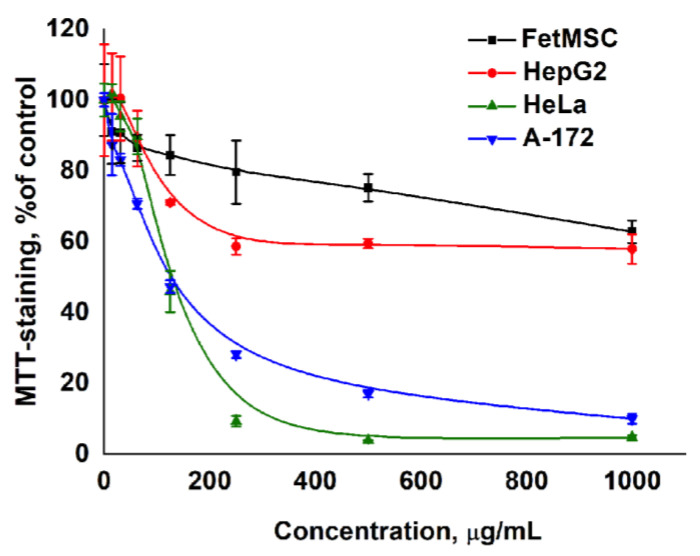
Effect of *Sequoia* extract on the viability of normal and tumor cells. HepG2—human hepatocellular carcinoma cell line; HeLa—“immortal” cervical cancer cell line; FetMSC—mesenchymal stem cells from the bone marrow of a 5–6-week-old human embryo; A172 A-172 —human glioblastoma cell line. Cells stained with MTT, with the control taken as 100%. On the graphs, the x-axis represents the final concentration of dry matter in the culture medium, and the y-axis represents the intensity of MTT staining as a percentage of the control.

For assessing the cytotoxic effects of complex extracts from the biomass obtained from callus tissue during the third cultivation cycle on the viability of cell cultures (FetMSC, HepG2, HeLa, and A-172), the MTT test was employed. Below are the dose-response curves for normal (FetMSC) and tumor (HepG2, HeLa, and A-172) cells after 72 h of exposure to the extracts. The staining of cells with the MTT dye in the control was taken as 100%. The graphs plot the final concentration of the dry substance in the culture medium on the x-axis and the intensity of MTT staining as a percentage of the control on the y-axis (Figure 12).

For the callus extracts, IC50 values (the concentration that inhibits cell viability by 50%) were determined. The results indicated that the studied extracts were low in toxicity to normal FetMSC cells and HepG2 liver carcinoma cells but demonstrated high toxicity toward the immortal HeLa cervical cancer cell line and the human glioblastoma A-172 cell line.

## 4. Discussion

*Sequoia* plants are relict long-livers that significantly differ from other representatives of the plant world due to their gigantic size, which is undoubtedly determined by specific physiological, biochemical, and anatomical features [36]. From the histochemical studies presented above, it follows that the studied *Sequoia* plants are characterized by a high ability to form secondary metabolites of various natures. This localization of polyphenols and terpenoids, including in *Sequoia*, is generally determined by their physiological functions. Most likely, the presence of polymer forms in the protective tissues of *Sequoia* provides the plants with protection against mechanical damage and pathogens, while soluble phenolic compounds in the underlying tissues may play the role of reserve and physiologically active substances, including those with anti-radical and cryoprotective properties (Figure 2). Previously, when studying the localization of polyphenols in plant tissues, researchers in many cases noted the accumulation of flavonoids mainly in the epidermal layers of leaves [37,38,39]. This dependence of the distribution of phenolic compounds in leaf cells has also been shown for tropical woody plants of mangrove forests [40]. At the same time, in plants, polymer phenolic compounds represented by lignin were predominantly localized in the cell walls of conductive tissues [41].

It is important to note the unique physiological feature of *Sequoia* plants in their ability to “combine” gigantic sizes, a high growth rate, and a long lifespan with simultaneous regeneration and renewal of young shoots in response to stress factors, which is undoubtedly ensured by a rich spectrum and plasticity of secondary (phenolic) metabolism. *Sequoia* leaves were characterized by the accumulation of metabolites in transfusion tissue cells. That is, they are confined to those structural elements of the tissue (mechanical fibers and xylem vessels) in which, among other things, the lignification process takes place [41,42]. This tendency in the localization of phenolic compounds is characteristic of many plants. In particular, it was observed in the stems of *Linum usitatissimum* L., *Populus trichocarpa* L., and *Corchorus capsularis* L. [43,44,45]. This served as the basis for the assumption that soluble phenolic compounds formed in plant parenchymal cells then move to the nearby walls of xylem vessels and mechanical tissue, where they are used to form a polymer—lignin. Lignin and other structural phenolic polymers accumulate in the cuticle and vascular system, ensuring vertical plant growth and resistance to drying and promoting water transport over long distances [41,46]. Phenolic compounds, such as salicylic acid, flavonols, tannins, (neo)lignans, or phytoalexins, which are “lighter” than lignin, act as chemical or olfactory signals to coordinate reactions to environmental factors and biotic interactions [47,48].

A special role is assigned to phenolic compounds, mostly flavonoids, in the photoprotection of plant tissues; accumulating in the cells of the epidermis and mesophyll (Figure 2), they prevent the pathological effects of UV rays on proteins and lipids, as well as on the photosynthetic and genetic machinery. Literature repeatedly reports that the initiation of the induction of secondary metabolite genes involved in protection against UV radiation is provoked by UV exposure [49,50].

The protective properties of secondary metabolites become particularly relevant for the foliage of the upper canopy, where high solar insolation is observed alongside water resource limitations, expressed in reduced leaf turgor as height increases [51,52]. The investment in the biosynthesis of substances with high antioxidant and protective properties, which polyphenols undoubtedly possess, underlies the high potential for improving the resistance of individual leaves to water stress as the height of the plant increases.

Understanding how the simultaneous biochemical and anatomical-morphological responses of *Sequoia* leaves regulate the changing hydrostatic potential with height can help resolve debates about the mechanisms that limit the maximum height of the tallest representatives of the plant world.

Researchers have repeatedly noted the special function of transfusion tissue, characteristic of gymnosperms, which participates in the bidirectional radial transport of metabolites between the leaf vein and the mesophyll cells. The role of transfusion tissue is associated with water storage, protection of the conductive system (xylem), low radial resistance, and efficient extraction of dissolved substances due to the increased surface area for contact between the vein and the mesophyll [53]. The increased volume of transfusion tissue in *Sequoia* contributes to enhanced hydraulic conductivity at the leaf level, thereby reducing radial resistance and increasing the maximum rate of photosynthesis [42]. In addition, the role of transfusion tissue of coniferous trees in frost resistance processes by controlling extracellular ice is known [54].

These physiological and regulatory properties of the developed transfusion tissue in *Sequoia* plants explain the widespread and abundant presence of secondary metabolites of phenolic and terpenoid nature in their cells (Figure 2). Our studies clearly support the idea that transfusion tissue, rich in secondary metabolites with high antioxidant properties, including flavonoids (catechins and proanthocyanidins), performs a protective function for the vessels during collapse/embolism, likely reducing the mortality of leaves (needles), branches, and tree tops during severe water stress.

Using specific histochemical reactions, we observed that starting from the earliest stages of the development of leaf primordia in the bud and subsequently in the leaves and stems at various stages of vegetative growth, there are special zones with increased localization of polyphenols (cuticle, stomata, epidermal, vascular, and transfusion tissues, parenchyma surrounding the vascular bundle, and albuminous cells; Figure 2). These polyphenol hyperaccumulator zones correspond to the structures that determine the hydraulic capacity of *Sequoia* plant tissues, influencing their ability to keep stomata open longer.

It has previously been shown that protection against UV-B radiation in plant tissues is associated with high concentrations of soluble phenolic compounds, such as flavonoids [38]. Such biochemical investments by *Sequoia* plants in low-molecular-weight and highly reactive secondary metabolites represented by flavanols may contribute to an increase in mitochondrial respiration rates [55,56]. All of this is reflected in the abundant localization of secondary metabolites, the biosynthesis of which is linked to carbohydrate metabolism products.

Special attention should be paid to the localization of flavanols—substances with pronounced antioxidant properties. As seen in Figure 2, they are an essential component of the cuticle, cell walls, and intercellular spaces of epidermal cells and stomata cells, further demonstrating the high reactivity of these low-molecular-weight compounds.

It is possible that the active biosynthesis and accumulation of terpenoids and polyphenols in the vascular and associated tissues also represent a certain adaptive component that helps the plant organism overcome stress factors that arise with increased height [57]. By localizing as a dense continuous layer around the perimeter of the leaf and stem, polyphenols contribute to the resilience of *Sequoia* against viral and fungal diseases as well as damage from animals, thus playing a role in the mechanisms that ensure the remarkable longevity of these giant relict plants, as has been reported in the literature [58,59,60].

The formation of callus tissue on the *Sequoia* explant is zoned and depends not only on various external factors (the composition of the nutrient medium and the lighting regime) but also on physiological and biochemical factors, primarily the presence of phenolic compounds and their localization in the primary explants. Comparing the obtained data led to the conclusion that the callus tissue of *Sequoia* formed in areas of the explant where the localization of phenolic compounds is minimal. This is likely because a high content of phenolic compounds leads to the inhibition of many physiological processes during dedifferentiation.

Furthermore, histochemical studies established that in callus cultures initiated from stem segments and grown on nutrient media in darkness, the number of cells containing phenolic compounds was lower than in the callus also derived from stem segments but grown in the presence of light. This likely resulted in the rapid inhibition of tissue growth initiated in the light. This pattern was characteristic of both studied types of nutrient media. An increase in the biosynthesis of phenolic compounds in response to the presence of a light source has been repeatedly noted by researchers at various plant sites [61,62], including due to the formation of flavanols in green plant tissues—the most common polyphenols, the biosynthesis of which is confined to chloroplasts [63]. This once again indicates that hyperaccumulation of polyphenols, in response to the presence of lighting, in callus tissue may be an indirect cause of inhibition of the growth of Sequoia callus culture [64]. We have previously observed a similar physiological and biosynthetic response to the presence of lighting when working with other representatives of relict gymnosperms—plants of the genus Taxus [65].

It should be noted that a significant feature of the Sequoia cell cultures is that a high level of polyphenol accumulation does not hinder their rapid growth, unlike other representatives of gymnosperms. Isolated studies in this direction show that S. sempervirens callus cultures not only retain their original biosynthetic ability with respect to compounds of phenolic and terpenoid nature but also enhance their biosynthesis in response to a change in the balance of growth regulators [21,66]. However, some components previously present in the “maternal” intact tissues of the explant were no longer synthesized in the extracted lipid fraction from in vitro cultures [21].

Callus cultures initiated from the same plant species may possess different abilities to accumulate polyphenols, which is also reflected in their intracellular localization. The data on the localization of phenolic compounds in callus tissues in the zone of morphogenesis deserve special attention (Figure 9). In the tissue of the callus mass adjacent to the forming shoot, there is an accumulation of cells containing secondary metabolites, which further underscores the regulatory and protective roles of polyphenols.

As is known, polyphenols not only influence the growth processes of the plant organism as a whole [67] but also exhibit allelopathic and fungicidal properties. Previous researchers have suggested that the “extractive substances of the heartwood”, represented by numerous organic compounds, possess fungicidal, bactericidal, or insecticidal properties [21,58], which determine the durability and specific coloration of “redwood” wood. Our studies also demonstrate high fungicidal activity not only in extracts obtained from intact plants but also from callus cultures (Table 1). Additionally, research on the cytotoxicity of extracts from *Sequoia* callus cultures shows high levels of activity against the “immortal” cervical cancer cell line HeLa and the human glioblastoma line A172. This is consistent with the data of the earlier screening of methanol extracts of *Sequoia* sempervirens cones for the presence of potential anticancer agents [68].

When comparing the obtained results with the cytotoxicity of the drug Taxol (Paclitaxel), it should be noted that the drug also exhibits more toxic effects on the HeLa and A-172 cells while being less toxic to HepG2 cells. At the same time, Taxol has a much more pronounced cytotoxicity: the IC50 for susceptible cells is 3 ng/mL, and for resistant cells, it is 35 ng/mL. The extracts we studied, obtained from one gram of raw biomass of *Sequoia* callus cells, are significantly less cytotoxic than the pure known drug (approximately 1000 times) but nevertheless represent an interesting potential source of biologically active compounds with potential antitumor activity.

The results obtained once again demonstrate that the studied plants actively synthesize secondary compounds, including those of phenolic nature, which are involved in key physiological processes. The alteration of the internal organization of tissues under in vitro conditions affects the formation of secondary compounds while maintaining the growth characteristics and biosynthetic capacity of *Sequoia* tissues at a high level in vitro. The new data on the fungicidal activity and cytotoxicity of extracts from in vitro cultures of the evergreen *Sequoia* indicate that they may serve as a source of valuable specific biologically active substances for phytopharmacognosy.

## 5. Conclusions

The studied intact *Sequoia* plants and in vitro cultures initiated from them actively synthesize secondary compounds of terpenoid and phenolic nature, which take part in key physiological processes [69,70]. The localization of secondary compounds (phenolic and terpenoid in nature) in intact *Sequoia* plants used as explants for the initiation of cell cultures in vitro has been shown for the first time. It has been shown that secondary metabolites are predominantly localized in epidermal, parenchymal, and conductive tissues. The localization of secondary compounds in callus and suspension cultures cultivated under different in vitro conditions as producers of substances with high biological activity was characterized. Alterations in the internal architecture of tissues under in vitro circumstances influence the production of secondary chemicals; an enhancement in polyphenol biosynthesis was noted when cultured callus lines under a 16 h photoperiod. In vitro cell cultures cultivated without light exhibited robust growth characteristics and maintained a high level of biosynthetic capability. New data have been obtained on the fungicidal activity of extractive substances of intact tissues and in vitro cultures of *Sequoia*, which successfully inhibit the growth of *F. oxisporum* mycelium. The cytotoxicity of extracts from in vitro evergreen *Sequoia* cultures suggests that they may be a potential source of particular, physiologically active compounds for phytopharmacognosy. Despite their minimal toxicity to normal FetMSC cells, *Sequoia* extracts effectively inhibit the “immortal” cervical cancer cells HeLa and the human glioblastoma A-172 line.

## Figures and Tables

**Table 1:** Characteristics of callus tissue obtained on MS and WPM media.

Indicators	Callus	
	MS	WPM
**Diameter growth (cm)**	0.92	1.05
**Growth index (*I*)**	1.46	1.53
**Specific growth rate (μ)**	0.030	0.031

**Table 2:** Results of studies on the fungicidal activity of extract samples against *Fusarium oxisporum* L. after 21 days of cultivation (average diameter, cm).

Concentration of Extract, mg/L										
K	50	100	150	200	250	300	350	400	450	500
Extracts from the shoots of *Sequoia* during the winter growing period										
9.0 ± 0.4	9.0 ± 0.4	9.0 ± 0.4	8.7 ± 0.4	8.4 ± 0.3	8.3 ± 0.3	7.7 ± 0.3	6.2 ± 0.3	4.8 ± 0.3	4.3 ± 0.2	3.7 ± 0.2
Extracts from callus tissue										
9.0 ± 0.4	9.0 ± 0.4	9.0 ± 0.4	9.0 ± 0.4	9.0 ± 0.3	8.8 ± 0.3	8.6 ± 0.3	7.5 ± 0.3	6.9 ± 0.3	6.1 ± 0.3	5.8 ± 0.3

## Data Availability

Data are contained within the article.
